# Wiped out by an earthquake? The ‘extinct’ Taiwanese swallowtail butterfly (Lepidoptera, Papilionidae) was morphologically and genetically distinct

**DOI:** 10.1371/journal.pone.0310318

**Published:** 2024-11-20

**Authors:** Vazrick Nazari, Shen-Horn Yen, Yu-Feng Hsu, Galina Shapoval, Nazar Shapoval, Valentina Todisco

**Affiliations:** 1 Department of Biology, University of Padova, Padova, Italy; 2 Department of Biological Sciences, National Sun Yat-Sen University, Kaohsiung, Taiwan, Republic of China; 3 Department of Life Science, National Taiwan Normal University, Taipei, Taiwan, Republic of China; 4 Department of Karyosystematics, Zoological Institute, Russian Academy of Sciences, Saint-Petersburg, Russia; 5 Department of Environment and Biodiversity, University of Salzburg, Salzburg, Austria; University of Mississippi School of Pharmacy, UNITED STATES OF AMERICA

## Abstract

For the first time, we obtained for the first time a COI DNA barcode from museum specimens of the Old World swallowtail butterfly endemic to Taiwan, *Papilio machaon* ssp. *sylvina*, that has disappeared since the devastating Jiji earthquake in 1999 that shook Central Taiwan. We demonstrate that this population was not only phenotypically distinct, but also had a unique mitochondrial haplotype among all other Holarctic populations of *P*. *machaon*. The life history of *P*. *m*. *sylvina* from rearing experiments carried out in the 1990s is illustrated and discussed.

## Introduction

Old World Swallowtail (*Papilio machaon* Linnaeus, 1758) is a well-known butterfly that is widespread in the Palearctic region with numerous subspecies recognized [1]. In Taiwan, two subspecies are known to occur: the population in Matsu Islands is closely related to *P*. *m*. *schantungensis* Eller, 1936 [2] described from Shandong province in China, while the main Island of Taiwan hosted an endemic subspecies, *P*. *m*. *sylvina*.

*Papilio machaon “sylvia”* was described in 1930 by Japanese Lepidopterists Teiso Esaki and Tadao Kano from mainland Taiwan based on two males collected in 1921 at “Torotsuku in the vicinity of Musha” (Jingguan/Wushe, Nantou county, Ren’ai) [3]. They named it after the second highest mountain in Taiwan, Mt. Sylvia (now known as Xueshan). However, since the name *sylvia* Esaki & Kano, 1930 was preoccupied by *Papilio sylvia* Fabricius, 1775, the replacement name *sylvina* was later proposed by Hemming [4]. Other names used for the mainland Taiwanese population, including *formosanus* Eller 1939 (nomen nudum) and *sylviae* Seyer 1976 (incorrect subsequent spelling) are invalid. Seyer [5] also placed *P*. *machaon* from Fujian under *sylvina*, and Moonen [6] considered *sylvina* as a subspecies of the Japanese *P*. *hippocrates* C. & R. Felder, 1864.

The life history of *P*. *m*. *sylvina* was first illustrated by Lin (1994) in the little-known book, “*The Butterflies and Nature of Taiwan*” [7]. The butterfly was endemic to and highly localized in the Central Mountain Range in Taiwan, mainly in mid- to high elevations (1000 to 2500m). Its larvae fed on *Peucedanum formosanum* (Umbelliferae), it had at least three generations annually and adults emerged mainly from May through September [5, 8, 9].

The catastrophic earthquake that shook Taiwan on 21 September 1999, known as the “Jiji earthquake”, resulted in multiple landslides in the habitat of *P*. *m*. *sylvina* that permanently altered the landscape in many parts of the Island. Efforts to restore the vegetation in those areas is ongoing [10]. At that time of the year, *P*. *m*. *sylvina* populations would have been at pupal stage and attached to the hostplants that were completely buried under the mudslides. Since then, despite exhaustive searches, *P*. *m*. *sylvina* has not been seen again, even though its larval hostplant still persists in the areas where it used to frequent. It is thus presumed extinct [1, 9, 11]. If this is truly the case, it may be the first known instance of an earthquake resulting in the complete demise of a local butterfly population.

The compilation of a Red List for Taiwanese butterflies is still ongoing, but for the time being *sylvina* has been given a “Critically Endangered” (CR) status (I-Ching Chen, pers. comm.). Many questions persist about the faith of *sylvina* but also about its taxonomic status, range, and genetic affinity with respect to other conspecific populations across its range. To compare the genetic characteristics of this population with others across the range of *P*. *machaon* in the Palaerctic region, we obtained a COI barcode sequence from a specimen of *P*. *m*. *sylvina* reared by SHY in 1995. We also compared the life history of this subspecies with that of *P*. *m*. *schantungensis* from the Matsu Islands of Taiwan.

## Materials and methods

**Fig 1:**
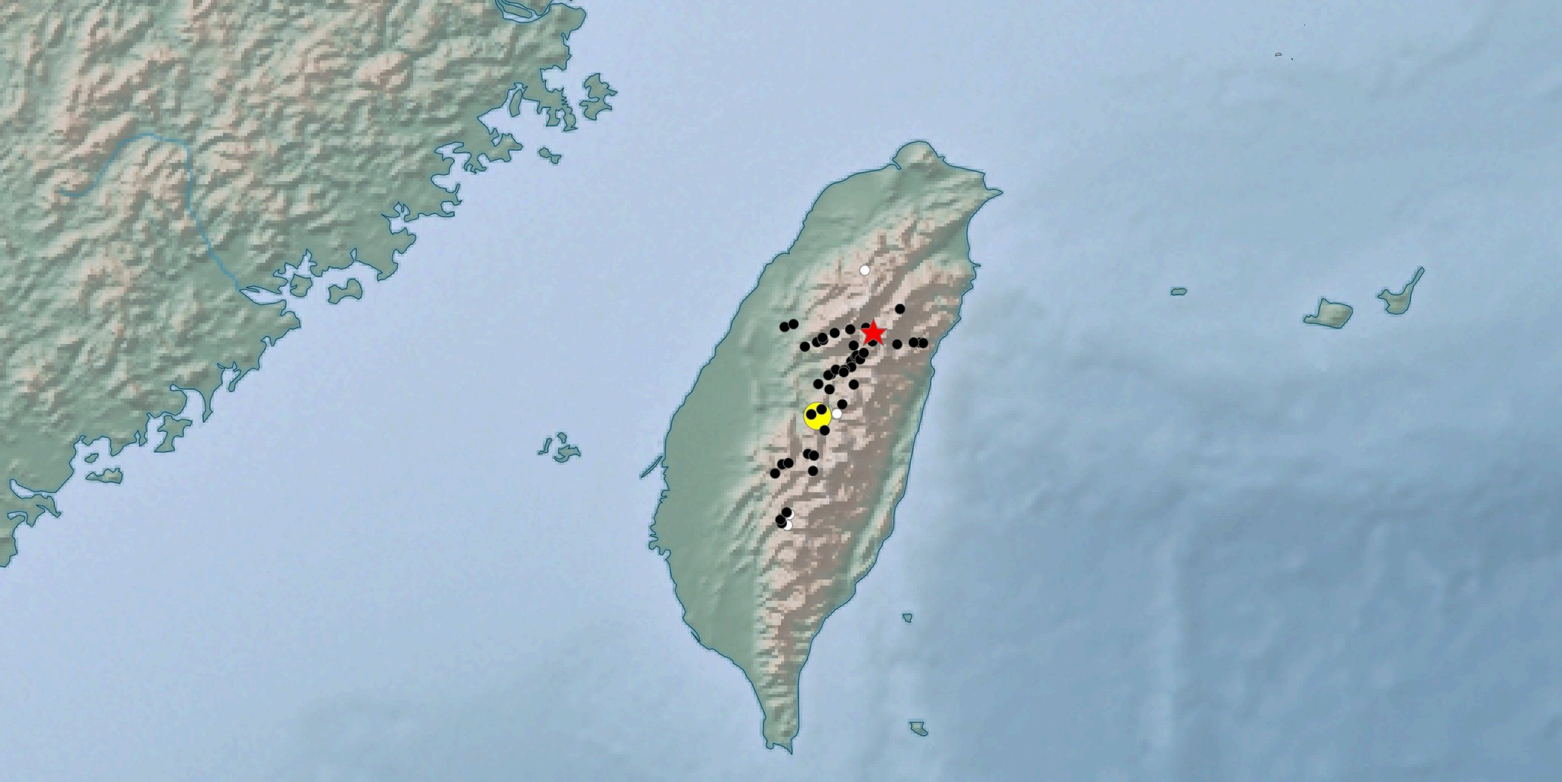
Historical occurrence records of *P*. *m*. *sylvina* in Taiwan. The white circles are approximate locations, the red star shows the type locality of *sylvina*, and the yellow circle marks the epicenter of 1999 Jiji earthquake. For locality details see S1 Table. Map created using simplemappr.net (Shorthouse 2010; in Public Domain).

Historical occurrence data for *P*. *m*. *sylvina* were summarized from past publications [9, 12, 13] and projected on a map using the Public Domain tool SimpleMappr [14] (S1 Table (which includes citations for References [15, 16]); Fig 1). Genitalia preparations of single individuals of both sexes of *P*.*m*. *sylvina* and *P*.*m*. *schantungensis* were made in the Department of Life Science, National Taiwan Normal University (Taipei) using standard methods. Dissections of genitalia were performed by removing the entire abdomen and placing it in 10% KOH at room temperature for 24 h to dissolve the soft tissue, then transferring it to cellusolve for another 24 h for descaling, before finally placing it in 70% ethanol for dissection. The dissected parts were preserved in 70% ethanol. Valvae of the male were spread in xylene for position fixing. All parts were slide mounted in euparal.

Legs were removed from two specimens of *P*. *m*. *sylvina* in the collection of SHY and processed in the Center for Biodiversity Genomics in Guelph (Canada) as well as in Zoological Institute, St. Petersburg (Russia), using primers and protocols described previously [17, 18]. Voucher specimens and rearing artefacts are kept in SHY collection in the campus of National Sun Yat-Sen University (Kaohsiung). A COI barcode dataset with the total length of 658 basepairs was then created to include additional populations of *P*. *machaon* across the Palearctic region [1, 19] as well as several sister species (Table 1). Published COI sequences were retrieved from GenBank and BOLD (www.boldsystems.org), and thirteen new sequences of various *P*. *machaon* subspecies (accessions PP865965 –PP865977) were submitted to GenBank (Table 1). Detailed collection data, images, and COI barcode sequences for all specimens used in this study are publicly available in the BOLD dataset “DS-SYLVINA” (dx.doi.org/10.5883/DS-SYLVINA).

**Table 1:** Material examined and GenBank accessions.

Taxon	Sample ID	Accession	Locality
*Papilio zelicaon*	NVG-2482	MW136708	USA: California, San Diego County, Otay Mountain
*Papilio polyxenes*	NVG-14113A01	MW136698	USA: California, San Bernardino County, Ivanpah Mountains
*Papilio hospiton*	not given	AF044009	Italy: Sardinia
*Papilio everesti*	DNAcdb0068	PP865966	China: Tibet, Nyalam
*Papilio hippocrates sachalinensis*	DNAwth031	PP865976	Russia: S. Sakhalin Isl.,10 km SW from Aniva city, Malinka village
*Papilio hippocrates hippocrates*	UASM 9900078	AY457593	Japan
*Papilio joanae*	FS312	KJ363192	USA: Missouri, Benton County, W of Warsaw
*Papilio brevicauda*	FS319	KJ363193	Canada: Newfoundland, Fishell
*Papilio machaon sylvina*	PAP113B	PP865969	Taiwan
*Papilio machaon schantungensis*	HSU03	PP865968	Taiwan: Lienchiang County, Matsu (Mazu) Islands, Tiebao, Nangan
*Papilio machaon schantungensis*	PAP065	PP865972	China: Fujian, Yongchun County, Yudou
*Papilio machaon schantungensis*	PAP073	PP865977	China: Shandong, Qingdao
*Papilio machaon asiatica*	LOWA-NL-5	PP865975	Nepal: Jumla
*Papilio machaon orientis*	AC-VT007	PP865971	Russia: Buryatiya Republic, Ulan Ude
*Papilio machaon ladakensis*	PAP011	PP865965	India: Ladakh, Leh district, Namshang
*Papilio machaon ussuriensis*	AC-SP1628	PP865974	Russia: Primorskiy Kray, S. Ussuri, Khasan District, Barabash
*Papilio machaon aliaska*	DNA 2300	FJ808896	Canada: Alberta, Peace River, S of Bear Canyon
*Papilio machaon verityi*	GCB01	PP865967	Vietnam: Dongvan
*Papilio machaon archias*	PAP074	PP865970	China: Sichuan, Ganzi, Mt. Gonggashan
*Papilio machaon machaon*	MM00727	HM871158	Finland: Ostrobottnia ouluensis, Liminka
*Papilio machaon kamtschadalus*	PAP056XR	PP865973	Russia: Kamchatskiy Kray, Esso village
*Papilio machaon centralis*	not given	HM243594	China: Tianshan Mountains

Genetic distances were calculated using MEGA 11.0.8 [20]. A Maximum Likelihood analysis was performed using the IQTree webserver [21] under default parameters with 1000 bootstrap alignments. Bayesian analysis was carried out with BEAST [22]. The tree was calibrated using several dates previously inferred for subgenus *Papilio* [23]: The root of the *P*. *machaon* subgroup (6.7 mya), the split between *P*. *hospiton* and the rest (5.1 mya), the split between *P*. *zelicaon* and *P*. *polyxenes* (3 mya), and the split between *P*. *brevicauda+P*. *joanae* and *P*. *machaon* (2.1 mya). The calibration points were set with uniform distributions and under an optimized relaxed clock and GTR substitution models. The analysis was allowed to run for 20 million generations and was repeated multiple times to check for convergence and stationarity, and the results were tested using TRACER 1.7.1 [24]. The resulting consensus tree was viewed in FigTree 1.4.4 [25] and edited using the open source software Inkscape (inkscape.org).

## Results

### Adult morphology

**Fig 2:**
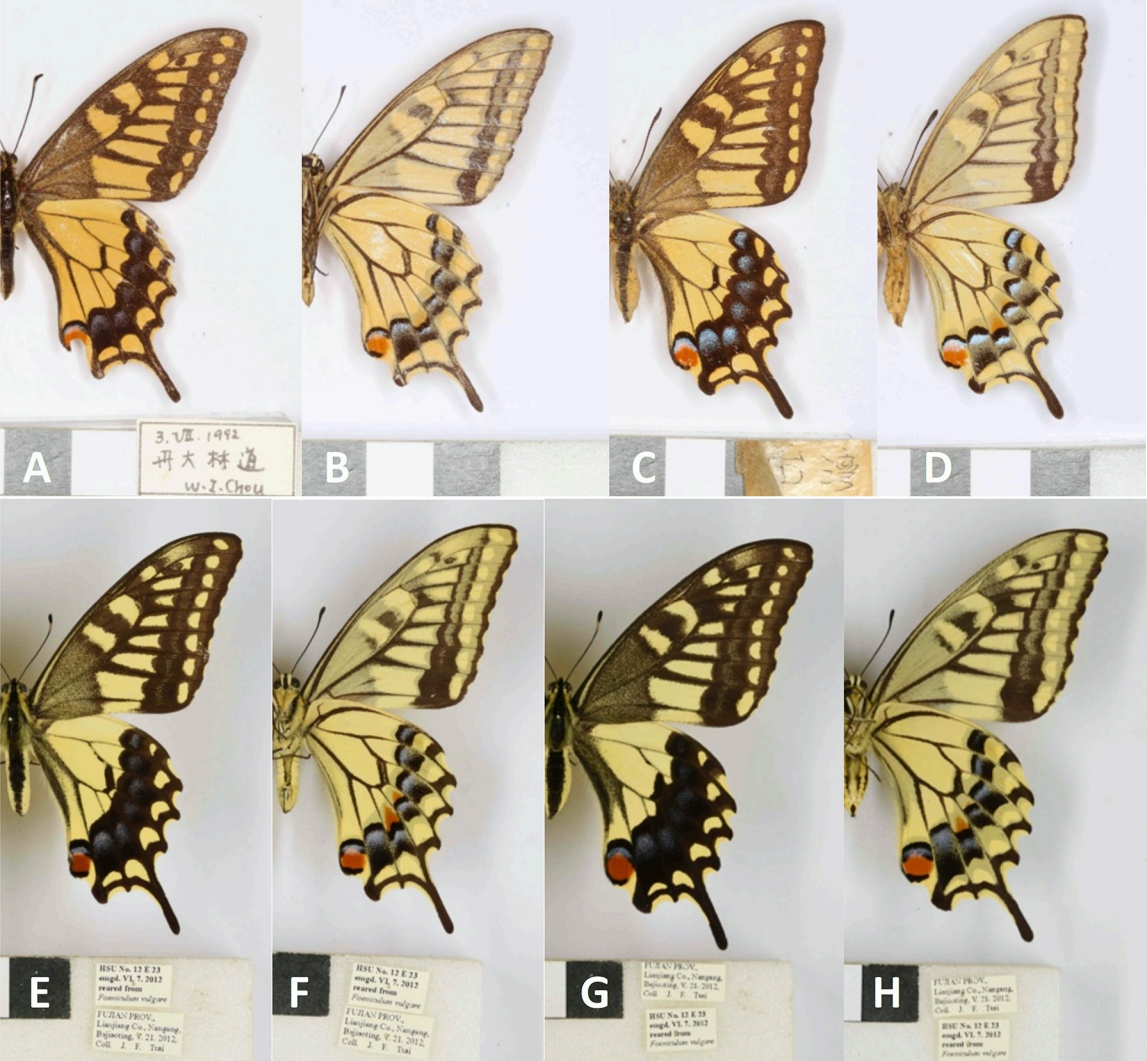
Collection specimens of *P*. *m*. *sylvina* and *P*. *m*. *schantungensis* from Taiwan. Upperside, underside: **A,B)** *P*.*m*. *sylvina* ♂, Taiwan: Nantou Co., Xinyi, Danda Forest Trail, VII. 3. 1992, Coll. W. I. Chou; **C,D)** *P*.*m*. *sylvina* ♀, “Taiwan”, specimen collected by the late Chin-King Yu, deposited at the Mu Sheng Insect Museum at Puli; **E,F)** *P*.*m*. *schantungensis* ♂, Fujian: Lianjiang Co., Nangang, Bajiaoting, V.21.2012, coll. J.F. Tsai, reared on *Foeniculum vulgare*, VI.8.2012, HSU No. 12E23; **G,H)** *P*.*m*. *schantungensis* ♀, same data as male. Scale bars = 1 cm. Photos by YFH.

**Fig 3:**
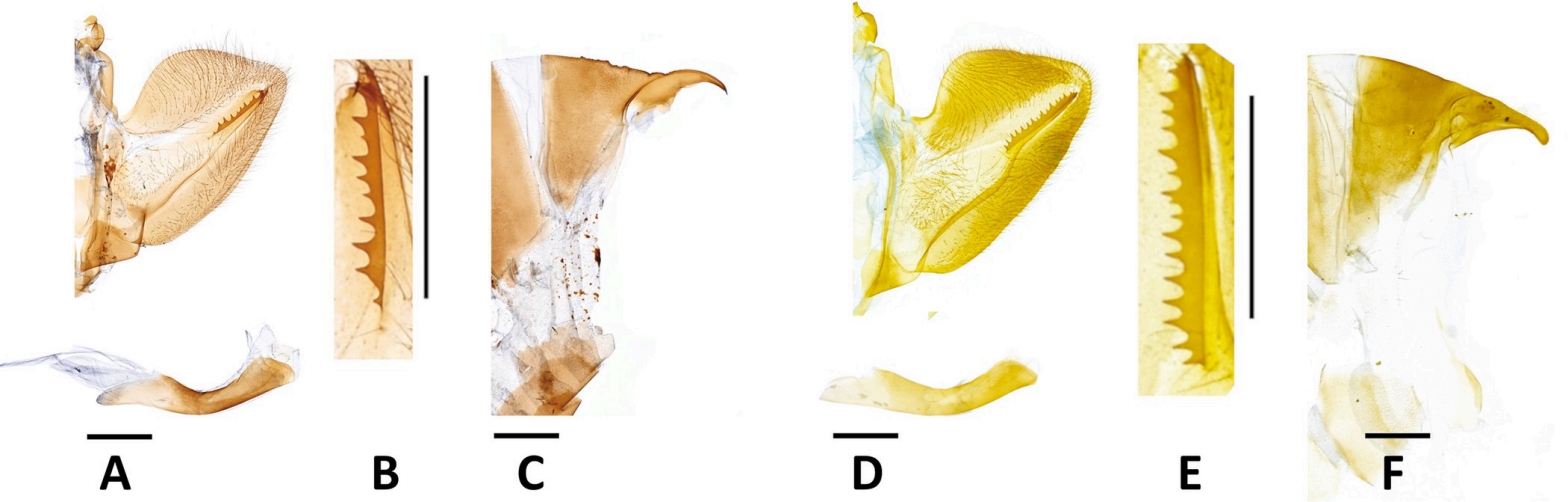
Male genitalia. A,D: right valvae and phallus, B,E: close-up of the harpe, C,F: side view of tergite VIII and superuncus. **A–C)** *P*. *m*. *sylvina*, Taizhong City [= Taichung Co.], Heping, Deji, VI.16.1990, H. Y. Lee; genitalia preparation JYL150. **D–F)** *P*. *m*. *schantungensis*, Lianjiang Co., Nangan, Bajiaoting, V. 21. 2012, reared from *Foeniculum vulgare*, emgd. VI. 7/8. 2012, HSU 12E23, J. F. Tsai; genitalia preparation JYL151. Scale bar = 1 mm. Photos by Jia Yuan Liang.

**Fig 4:**
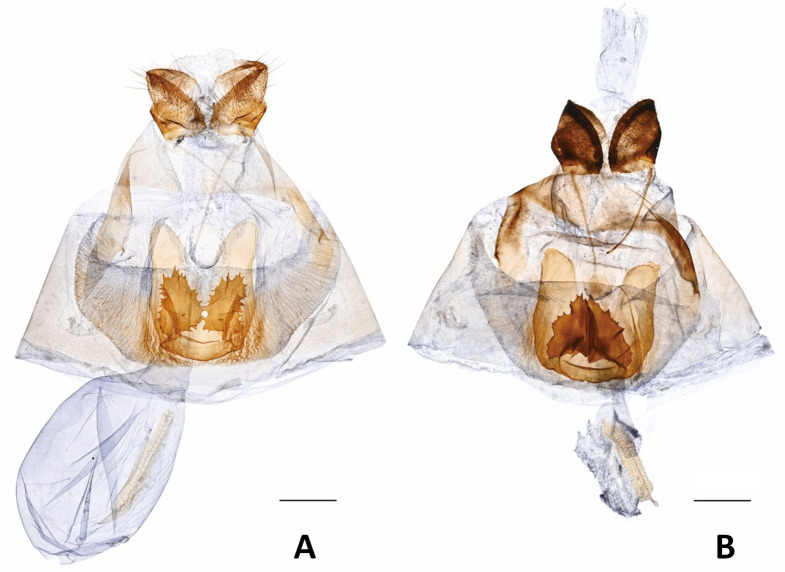
Female genitalia. **A)** *P*. *m*. *sylvina*, Taiwan: Taizhong City [= Taichung Co.], Heping, Guguang, VII.24.1993 (S. Miyazaki). **B)** *P*. *m*. *schantungensis*, Taiwan, Lianjiang Co., Nangan, Bajiaoting, V. 21. 2012, reared from *Foeniculum vulgare*, emgd. VI. 7/8. 2012, HSU 12E23 (J. F. Tsai). Scale bar = 1 mm. Photos by Jia Yuan Liang.

Compared to individuals of *P*. *m*. *schantungensis* from the Matsu Islands of Taiwan and others on the mainland, *P*. *m*. *sylvina* adults were usually smaller and had a much deeper brownish-yellow ground-color, well-developed black scaling on the veins on the upperside of the wings, a narrower dark submarginal band and a wider yellow median band on the upperside of the forewings, and a narrow dark submarginal band on the upperside of the hindwings positioned far from the disco-cellular apex (Fig 2). In the examined male genitalia of *sylvina*, the harp is shorter with ~11 teeth (longer with ~17 teeth in *schantungensis*), and the distal edge of super tegumen near junction with proximal end of supra-uncus is rounded and bulged with a pointed apex (straight in *schantungensis* with a dull apex) (Fig 3). In the female genitalia, ductus bursa is thicker; signum is narrower and more elongate; sclerotized extensions of lamella postvaginalis are wider and have a different pattern of serration (Fig 4).

### Biology

While the larvae of *P*. *m*. *schantungensis* feed on a variety of host plants (Apiaceae: *Angelica decursiva*, *A*. *hirsutiflora*, *A*. *tarokensis*, *Apium graveolens*, *Coriandrum sativum*, *Cryptotaenia canadensis*, *Daucus carota*, and *Peucedanum formosanum*) [26–28], the larvae of *P*. *m*. *sylvina* were recorded only on *Peucedanum formosanum* under natural condition [7, 9]. Records of other host plants for *P*. *m*. *sylvina* (e.g. *Angelica dahurica*, *A*. *biserrata*, *A*. *hirsutiflora*, *A*. *tarokensis*, *A*. *morii*, *Apium graveolens*, *Coriandrum sativum*, *Cryptotaenia canadensis*, *Daucus carota*) [27–30] have been copied from literature pertaining to the Japanese or Chinese subspecies of *P*. *machaon* and are erroneous. The preferred nectar plants for the adults of *P*. *m*. *sylvina* included *Lespedeza lucidum*, various *Gypsophila*, *Malanthus*, *Astragalus* spp., and *Asparagus* spp. that still grow on the cliffs in the Central Mountain Range in Taiwan.

**Fig 5:**
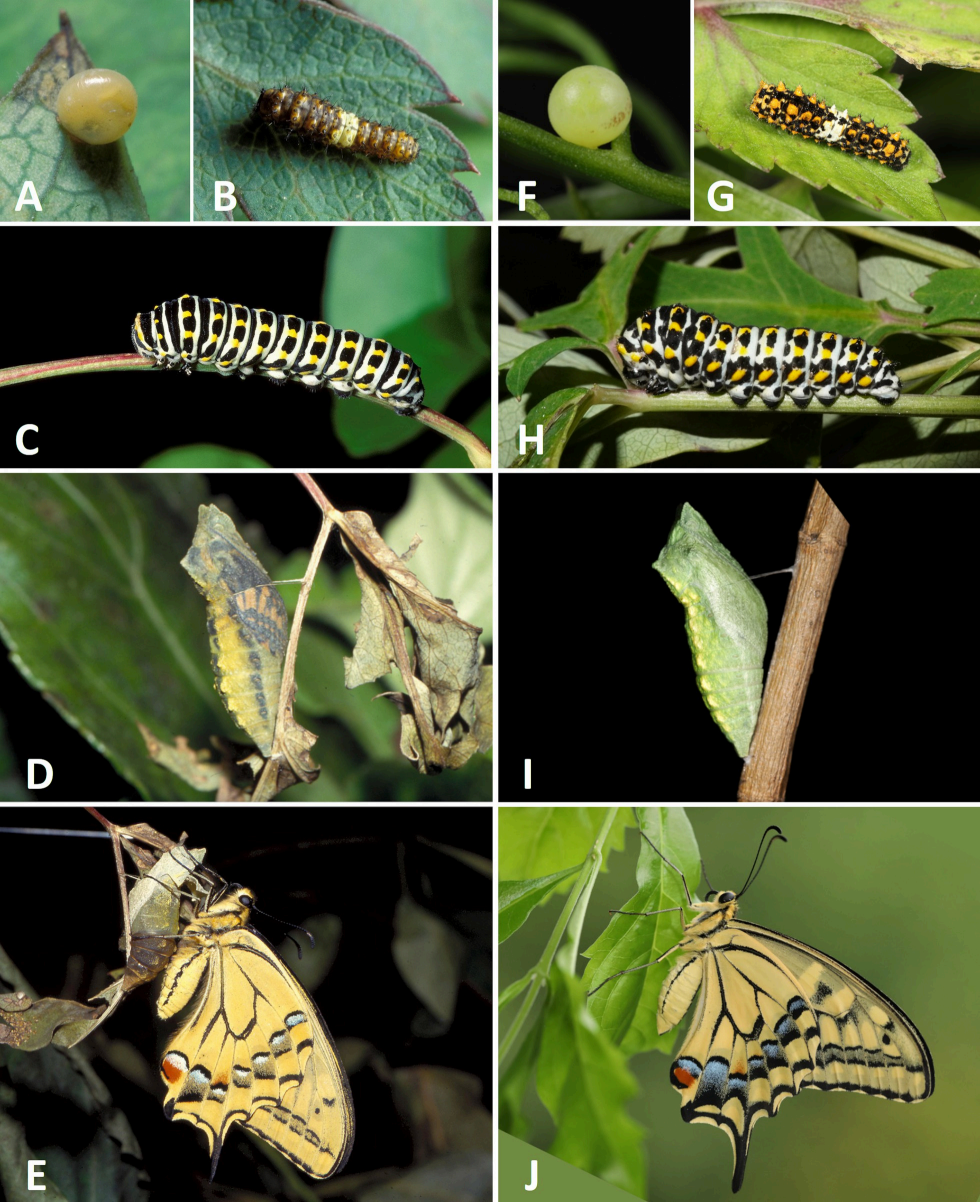
Life history of *P*. *m*. *sylvina* (A–E) and *P*. *m*. *schantungensis* (F–J) from Taiwan. A,F) ova, B,G) second instar larvae, C,H) fourth instar larvae, D,I) pupae, E,J) freshly emerged adults. Photos by JL Jean (A,B), SHY (C–E), and YFH (F–J).

Comparing the early life stages of *P*. *m*. *sylvina* (eggs, caterpillars and pupae) with those of *P*. *m*. *schantungensis* (Fig 5), even though minor differences could be observed, we did not find any unique characters that could be considered outside of the normal range of individual variation known in *P*. *machaon*. The female attached eggs to the underside of leaves and stems of host plants. After the larvae hatched, they moved to the surface of the host leaves, and then usually pupated on the still objects near the host plant, or more rarely on the back of the host leaves and stems [7]. Adult butterflies had a fast flight and were highly mobile. They often flew away from their breeding grounds and visited places at lower or higher altitudes. According to Lin [7], the adult *P*.*m*. *sylvina* were extremely sensitive to yellow objects and were often attracted by the yellow reflective lenses on roadsides.

### DNA analysis

**Fig 6:**
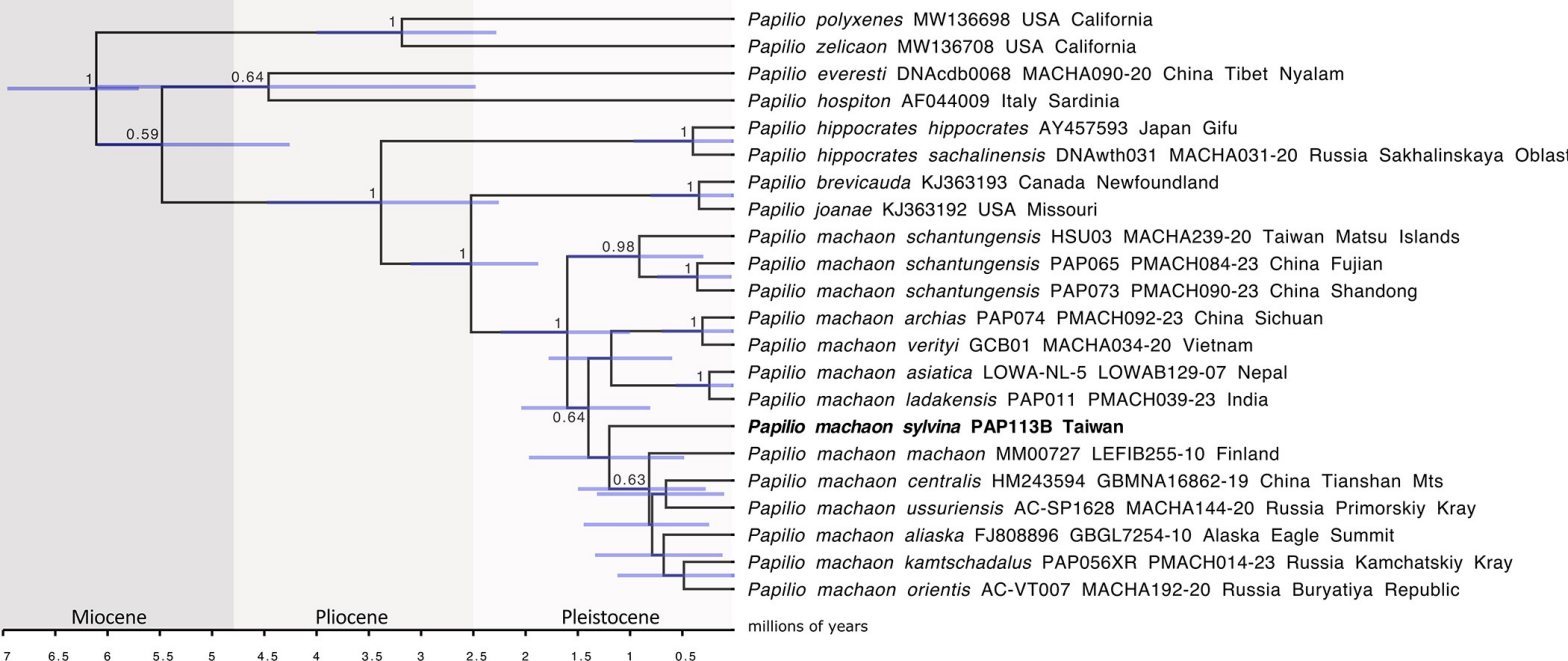
BEAST tree of COI barcodes for selected *Papilio* species. Node values show Bayesian Posterior Probabilities for supported nodes (>0.5)(for median node ages see S1 Fig).

A 610-basepairs mitochondrial COI barcode was successfully retrieved from one of the two samples of *P*. *m*. *sylvina*, and the results obtained in the two laboratories (Canada and Russia) were identical. The COI barcode of *P*. *m*. *sylvina* is 0.82% distant from those of the Matsu Island population, and 0.49–1.16% (mean: 0.79%) distant from other populations of *P*. *machaon* across its range (Table 2). According to the Barcode Index Number (BIN) system on BOLD, our sequence of *P*. *m*. *sylvina* falls within the large Holarctic cluster of *P*. *machaon* (BOLD:AAA5810) indicating that it is part of the larger variation within *P*. *machaon*. In our phylogenetic analysis, *P*. *m*. *sylvina* appeared nested well within the *P*. *machaon* clade and as sister to a group including populations from Eastern and Northern Palearctic region and Alaska (Fig 6). Our results also reject previous hypotheses posed by Seyer [5] that the Fujian populations belong to *P*. *m*. *sylvina*, and by Moonen [6] that *sylvina* should be considered a subspecies of *P*. *hippocrates* C. & R. Felder, 1864. Interestingly, the Matsu Islands population of *P*. *machaon*, often referred to as ssp. *schantungensis*, showed a different haplotype compared to the topotypical ssp. *schantungensis* from Shandong as well as those in Fujian.

**Table 2:** K2P distances between taxa examined in this study.

Taxon	1	2	3	4	5	6	7	8	9	10	11	12	13	14	15	16	17	18	19	20	21	22
1. *Papilio zelicaon*																						
2. *Papilio polyxenes*	3.29																					
3. *Papilio hospiton*	3.60	4.08																				
4. *Papilio everesti*	4.74	4.74	3.13																			
5. *Papilio hippocrates sachalinensis*	4.41	4.08	3.45	3.45																		
6. *Papilio hippocrates hippocrates*	4.58	4.25	3.61	3.29	0.15																	
7. *Papilio joanae*	4.91	4.75	3.78	3.13	2.17	2.01																
8. *Papilio brevicauda*	4.75	4.58	3.62	2.97	2.01	1.86	0.15															
**9. *Papilio machaon sylvina***	4.07	4.06	3.56	3.21	2.17	2.17	1.83	1.66														
10. *Papilio machaon schantungensis*	4.41	4.08	3.61	3.45	2.01	2.01	1.54	1.38	0.82													
11. *Papilio machaon schantungensis*	4.74	4.57	3.93	3.60	2.48	2.48	2.17	2.01	0.99	0.61												
12. *Papilio machaon schantungensis*	4.41	4.24	3.93	3.93	2.48	2.48	2.17	2.01	0.83	0.61	0.30											
13. *Papilio machaon asiatica*	4.75	4.74	4.26	3.93	2.80	2.80	2.49	2.33	1.16	1.23	1.54	1.54										
14. *Papilio machaon orientis*	4.09	4.08	3.61	3.28	1.85	1.85	1.54	1.38	0.49	0.61	0.92	0.92	0.92									
15. *Papilio machaon ladakensis*	4.58	4.57	4.10	3.77	2.64	2.64	2.33	2.17	0.99	1.07	1.38	1.38	0.15	0.77								
16. *Papilio machaon ussuriensis*	4.25	4.24	3.77	3.12	2.01	2.01	1.70	1.54	0.66	0.76	1.07	1.07	1.08	0.15	0.92							
17. *Papilio machaon aliaska*	4.25	4.24	3.45	3.12	2.01	2.01	1.70	1.54	0.66	0.76	0.76	1.07	1.08	0.15	0.92	0.30						
18. *Papilio machaon verityi*	4.75	4.74	4.26	3.93	2.48	2.48	2.17	2.01	0.99	0.92	1.23	1.23	1.23	0.61	1.08	0.77	0.77					
19. *Papilio machaon archias*	4.58	4.57	4.10	3.77	2.32	2.32	2.01	1.85	0.83	0.76	1.07	1.07	1.08	0.46	0.92	0.61	0.61	0.15				
20. *Papilio machaon machaon*	3.92	3.92	3.45	3.12	2.01	2.01	1.38	1.23	0.66	0.76	1.07	1.07	1.08	0.15	0.92	0.30	0.30	0.77	0.61			
21. *Papilio machaon kamtschadalus*	4.09	4.08	3.61	3.28	1.85	1.85	1.54	1.38	0.49	0.61	0.92	0.92	0.92	0.00	0.77	0.15	0.15	0.61	0.46	0.15		
22. *Papilio machaon centralis*	4.25	4.24	3.77	3.45	2.01	2.01	1.70	1.54	0.66	0.76	1.07	1.07	1.08	0.15	0.92	0.30	0.30	0.77	0.61	0.30	0.15	-

The taxa *verityi* Fruhstorfer, 1907 and *archias* Fruhstorfer, 1907 have recently been recognized as good species separate from *P*. *machaon* primarily based on their morphological characteristics [1], even though the preliminary COI data available to these authors did not support such a designation at least for *verityi*. Here we suggest that, in order to preserve the monophyly of *P*. *machaon* and pending verification by additional data, it is best to maintain these two names for now as subspecies of *P*. *machaon*.

## Discussion

Extinction is a natural phenomenon, but natural disasters are rarely known to have severely threatened the survival of butterfly species. One example is the Schaus Swallowtail *Papilio aristodemus ponceanus*, frequently threatened by hurricanes in the Florida Keys [31, 32]. However, as far as we know, the case of *P*. *m*. *sylvina* may be the first and only documented instance of extinction of a butterfly following an earthquake.

Taiwan was formed approximately 4 to 5 million years ago as a consequence of oceanic plate subduction at a complex convergent boundary between the Philippine Sea Plate and the Eurasian Plate (“The Penglai Orogeny”) [33, 34]. The Island fully separated from the mainland upon the formation of the Okinawa Trough and the Taiwan Strait, dated at 1.552 ± 0.154 Ma [35, 36]. This date is consistent with the divergence time inferred in our study for the split of the last common ancestor of *P*. *m*. *sylvina* from the continental *P*. *machaon* (mean: 1.4 mya), and was likely the vicariance event that resulted in the isolation of *P*. *m*. *sylvina* in the highlands of Taiwan (Fig 6).

The formation of the Central Mountain Range of Taiwan about 2.5–1 Mya [33, 37] resulted in diversification of the topography and climate and boasted a rich biodiversity. The composition of species inhabiting the lowlands and highlands of Taiwan are rather different: While the fauna in the lower altitudes generally show greater affinities with south Asian and tropical regions, the mountain species are principally Eurasian [38]. Repeated connection and disconnection of Taiwan from mainland China and Ryukyu Islands during the late Pleistocene, influenced by glacial-interglacial cycles that affected the sea level in the Taiwan Strait [39–41] provided opportunities for insular species, particularly those in the lowlands, to experience frequent isolation from, and secondary contact with, the continental mainland. The isolated highland fauna however was not particularly affected by these interglacial land bridges [35]. The ‘postglacial contraction hypothesis’ postulates that during the glaciations, taxa that were once widely distributed at lower elevations across Taiwan were driven to higher elevations when the climate changed at the end of the glaciations [42, 43]. However, isolation and ecological adaptation of *P*. *m*. *sylvina* to the higher altitudes prior to the Last Glacial Maximum is evident by the fact that continental populations of *P*. *machaon* with the most similar COI barcodes to ssp. *sylvina* appear to be not the ones on mainland China, but rather those from Northern Eurasia (Buryatia and Kamchatka, 0.49%) (Table 2). A similar pattern is also noted in *Ypthima* butterflies [44], saturniid moths [45], *Cylindera* and *Neolucanus* beetles [46, 47], *Carbula* bugs [48], funnel web-spiders [49] and *Trimeresurus* pit vipers [50].

Our study provides further evidence for the utility of mitochondrial DNA as a useful tool in elucidation of the phylogenetic affinities of extinct lepidopterans [51–53]. Whether the earthquake was the direct cause of its demise, or other factors had previously threatened the existence of *P*. *m*. *sylvina* is unclear. Effects of parasitism, pesticides, diseases, climate change, and commercial collecting prior to the earthquake have not been exhaustively studied and thus cannot be ruled out. It is clear however that this unique population was well on its evolutionary track to become its own distinct lineage as a separate species. It was, and still is, an important icon for Taiwan, as its image is imprinted on the personal ID cards of Taiwanese citizens [11]. Even though the butterfly has not been seen or collected since 1999, one can always hope that it still persists in the remote mountain regions in the Taiwan highlands.

## Supporting information

S1 Table Historical occurrence data for *P*. *m*. *sylvina*. (DOCX)

S1 Fig Median node ages inferred by BEAST analysis. (DOCX)
