# Correction: Wiped out by an earthquake? The ‘extinct’ Taiwanese swallowtail butterfly (Lepidoptera, Papilionidae) was morphologically and genetically distinct

**DOI:** 10.1371/journal.pone.0316738

**Published:** 2024-12-30

**Authors:** Vazrick Nazari, Shen-Horn Yen, Yu-Feng Hsu, Galina Shapoval, Nazar Shapoval, Valentina Todisco

The Funding statement for this paper is incorrect. The correct Funding statement is as follows: Open access funding for this study is provided by Università degli Studi di Padova. N.S. receives financial support from a grant from the Ministry of Science and Higher Education of the Russian Federation (no. 075-15-2021-1069) and from the State Research Project (no. 122031100272–3). G.S. receives financial support from the state assignment of the Ministry of Science and Higher Education of the Russian Federation (project no. FZMW-2023-0006).

## References

[1] Nazari V, Yen S-H, Hsu Y-F, Shapoval G, Shapoval N, Todisco V (2024) Wiped out by an earthquake? The ‘extinct’ Taiwanese swallowtail butterfly (Lepidoptera, Papilionidae) was morphologically and genetically distinct. PLOS ONE 19(11): e0310318. 10.1371/journal.pone.0310318 39565827 PMC11578470

