# Supplementary material for: Wiped out by an earthquake? The ‘extinct’ Taiwanese swallowtail butterfly (Lepidoptera, Papilionidae) was morphologically and genetically distinct
Source: PLoS One. 2024 Nov 20;19(11):e0310318. doi: 10.1371/journal.pone.0310318 (PMC11578470; doi:10.1371/journal.pone.0310318)
Supplement: S1 Table — (DOCX) [file pone.0310318.s001.docx]

**S1 Table.** Historical occurrence data for *P. m. sylvina*.

| **Source** | **Locality** | **Latitude** | **Longitude** |
| --- | --- | --- | --- |
| Kano (1930) | Type Locality (Torotsuku/Jingguan) | 24.2353289 | 121.2906006 |
| Yamanaka (1971) | 久良栖(松鶴) | 24.17971 | 120.97941 |
| Yamanaka (1971) | 佳保台 | 24.19113 | 121.01315 |
| Yamanaka (1971) | 八仙山 | 24.15452 | 120.91211 |
| Yamanaka (1971) | 明治溫泉/谷關溫泉 | 24.20415 | 121.00934 |
| Yamanaka (1971) | 埔里社(埔里東南部) | 23.94809 | 120.98645 |
| Yamanaka (1971) | 本部溪 | 24.00301 | 121.05714 |
| Yamanaka (1971) | タッタカ(立鷹/松崗) | 24.07414 | 121.16874 |
| Yamanaka (1971) | 三角峰 | 24.09898 | 121.19186 |
| Yamanaka (1971) | 見晴(清境) | 24.04201 | 121.16874 |
| Yamanaka (1971) | 追分(翠峰) | 24.10638 | 121.19875 |
| Yamanaka (1971) | 東埔 | 23.56276 | 120.92975 |
| Yamanaka (1971) | 樂樂 | 23.55372 | 120.9622 |
| Yamanaka (1971) | 露營地 **^1^** | - | - |
| Yamanaka (1971) | クヤニヤ **^2^** | 23.45552 | 120.74757 |
| Yamanaka (1971) | セラオカ(西拉歐卡) | 24.16779 | 121.42195 |
| Yamanaka (1971) | 濁水溪 **^3^** | - | - |
| Yamanaka (1971) | タロコ(太魯閣) **^4^** | - | - |
| Yamanaka (1971) | 土牛 | 24.2632 | 120.80018 |
| Yamanaka (1971) | 東勢 | 24.27885 | 120.84931 |
| Yamanaka (1971) | 大甲溪舊道 **^5^** | 24.575891 | 121.24364 |
| Yamanaka (1971) | 南湖大山 | 24.36198 | 121.43787 |
| Yamanaka (1971) | 獅子頭 | 23.99719 | 121.04175 |
| Yamanaka (1971) | 南山溪 | 24.02745 | 121.0832 |
| Yamanaka (1971) | 霧社 | 24.026 | 121.13123 |
| Yamanaka (1971) | トロック(靜觀) | 24.08475 | 121.21737 |
| Yamanaka (1971) | 桜ヶ峯 (櫻櫻峰) | 24.11989 | 121.23771 |
| Yamanaka (1971) | 紅葉(紅香) | 24.16114 | 121.18142 |
| Yamanaka (1971) | 合歡山 | 24.1826 | 121.28542 |
| Yamanaka (1971) | パーラン(巴蘭) | 24.01364 | 121.12674 |
| Yamanaka (1971) | 武界 | 23.91902 | 121.04843 |
| Yamanaka (1971) | 卓社大山 | 23.83609 | 121.11882 |
| Yamanaka (1971) | バクラス(巴庫拉斯) | 23.80898 | 121.00475 |
| Yamanaka (1971) | ヒノコン(西諾滾/雙龍) | 23.78086 | 120.94748 |
| Yamanaka (1971) | 望鞍 | 23.69245 | 121.02128 |
| Yamanaka (1971) | 阿里山 | 23.50533 | 120.78736 |
| Yamanaka (1971) | 祝山 | 23.51243 | 120.82196 |
| Yamanaka (1971) | 新高山(玉山) | 23.4688 | 120.95729 |
| Yamanaka (1971) | ガニ(雅爾) | 23.18068 | 120.78397 |
| Yamanaka (1971) | 濁水 | 23.19986 | 120.77757 |
| Yamanaka (1971) | ビビュウ(復興)～濁水 **^6^** | 23.208919 | 120.811088 |
| Yamanaka (1971) | ビビュウ(復興)～ラボラン(梅蘭) **^7^** | 23.229346 | 120.828001 |
| Yamanaka (1971) | ラボラン(梅蘭) | 23.24072 | 120.81139 |
| Yamanaka (1971) | 關山越道路 **^8^** | 23.169347 | 120.815692 |
| Yamanaka (1971) | 大斷崖 | 24.17687 | 121.54821 |
| Yamanaka (1971) | 錐麓 | 24.1742 | 121.56567 |
| Yamanaka (1971) | 合流 | 24.17774 | 121.51299 |
| Yamanaka (1971) | 紅頭嶼 (蘭嶼) **^9^** | - | - |
| Lin (1994) | 奧萬大 | 23.94545 | 121.18269 |
| Uchida (1995) | 石山溪 | 24.23054 | 121.07674 |
| Hsu et al. (2018) | Lishan (梨山) | 24.25862 | 121.2496 |
| Hsu et al. (2018) | Deji (德基) | 24.24951 | 121.16319 |
| Hsu et al. (2018) | Danda Forest Trail (丹大林道) **^10^** | 23.784514 | 121.089302 |
|  |  |  |  |
| Notes. |  |  |  |
| 1. literally meaning “camping ground”, with no further information to indicate where it is. | | | |
| 2. “Kuyaniya”; exact locality uncertain, but probably around the Cou tribe called “Tapangu”in Alishan area today. | | | |
| 3. One of the major rivers in Taiwan, with elevation ranging from 0 to over 3000m, no coordinate can be determined for this record. | | | |
| 4. The name of a large area relevant to a county ranging from 500 to over 3000m, no coordinate can be determined for this record. | | | |
| 5. midpoint of a 62 km-long ancient trail between 24.17570, 121.26230 and 24.97608, 121.22486. | | | |
| 6. midpoint between 23.21797, 120.84461 and 23.19986, 120.77757. | | |  |
| 7. midpoint between 23.21797, 120.84461 and 23.24072, 120.81139. | | |  |
| 8. midpoint of a 171 km-long ancient trail between 23.07449, 120.67048 and 23.26407, 120.96111. | | | |
| 9. in error; Yamanaka listed this locality citing Sonan [52] and Shirôzu [53], but this locality is not mentioned as a collecting site in both references. | | | |
| 10. midpoint of a 55 km-long trail between 23.78718, 121.01173 and 23.78181, 121.16687. | | | |
