# Supplementary material for: Wiped out by an earthquake? The ‘extinct’ Taiwanese swallowtail butterfly (Lepidoptera, Papilionidae) was morphologically and genetically distinct
Source: PLoS One. 2024 Nov 20;19(11):e0310318. doi: 10.1371/journal.pone.0310318 (PMC11578470; doi:10.1371/journal.pone.0310318)
Supplement: S1 Fig — (DOCX) [file pone.0310318.s002.docx]

**S2 Fig.** Median node ages inferred by BEAST analysis.
